# Impact of climatic factors on temporal variability of sand fly abundance in Sri Lanka: Longitudinal study (2018 to 2020) with two-stage hierarchical analysis

**DOI:** 10.21203/rs.3.rs-3098746/v1

**Published:** 2023-06-28

**Authors:** Sanath C Senanayake, Prasad Liyanage, Dulani R.K. Pathirage, M. F. Raushan Siraj, B. G. D. Nissanka Kolitha De Silva, Nadira D Karunaweera

**Affiliations:** Department of Parasitology, Faculty of Medicine, University of Colombo; Department of Research and Evaluation, National Institute of Health Sciences Kalutara; Department of Parasitology, Faculty of Medicine, University of Colombo; Department of Parasitology, Faculty of Medicine, University of Colombo; Center for Biotechnology, Department of Zoology, Faculty of Applied Sciences, University of Sri Jayewardenepura; Department of Parasitology, Faculty of Medicine, University of Colombo

**Keywords:** P. argentipes, rainfall, relative humidity, Distributed Lag Non-Linear modelling framework

## Abstract

**Background:**

Phlebotomine sand flies serve as vectors for leishmaniasis, a major health concern, but a neglected tropical disease. The risk of vector activity is governed by climatic factors that vary in different geographic zones in the country. Thus, we aimed to quantify the effect of climatic variables on sand fly vector activity in ten sentinel sites across Sri Lanka.

**Methods:**

Mean rainfall, ambient temperature, relative humidity, wind speed, soil temperature, evaporation, sunshine hours, and vector densities were recorded at monthly intervals in each location from March 2018 to February 2020. The association between weather variables and sand fly densities was analysed using a two-staged hierarchical procedure; Distributed Lag Non-Linear (DLNM) modelling framework and the DLNM method implemented in the R package *dlnm* (version number 2.4.6).

**Results:**

Moderate rainfall values up to 120 mm per month and increasing RH up to 82 at lag of 0 months along with increasing soil temperature and evaporation rate at lag of 2 months were associated with statistically significant increase in the sand fly activity. These associations were heterogeneous across study settings. Whereas increasing ambient and soil temperature, sunshine hours, evaporation rate appeared to reduce the sand fly activity homogeneously at lag of 0 month in all the study settings.

**Conclusions:**

The abundance of sand fly vectors varied in relation to selected climatic variables, either in real-time or with a time lag. This information can be utilized for predicting sand fly densities and for the development of effective strategies to prevent leishmaniasis transmission in specific settings.

## Background

The subfamily Phlebotomine (sand flies) includes as many as 800 species [1]. Sand flies are small (2 to 3mm in size) hairy hematophagous insects smaller than mosquitoes and live in warmer tropical and sub-tropical regions between 50°N and 40°S [2]. Sand flies can transmit several bacterial, viral, and parasitic diseases including leishmaniasis [1–3]. Leishmaniasis are a group of diseases caused by more than 20 *Leishmania* species parasites transmitted through the bites of infected female phlebotomine sand fly vectors [1]. More than 90 sand fly vectors are known to transmit the parasite. Different species of *Leishmania* cause different clinical manifestations ranging from self-limiting cutaneous lesions to life-threatening visceral disease [1]. The clinical outcome depends on the fine interplay between parasite characteristics, vector biology and host factors mainly the immune response [4]. Accordingly, the disease has three main forms; visceral (VL), the most serious form; mucocutaneous (MCL) the most disabling and cutaneous (CL), the most common [1]. It is estimated that between 0.7 to 1 million new cases of cutaneous leishmaniasis occur annually, ranking it third among neglected tropical diseases [5]. Although the disease is endemic in approximately a hundred tropical and sub-tropical countries, over 85% of new cases were reported only in ten countries: Afghanistan, Algeria, Brazil, Colombia, Iraq, Libya, Pakistan, Peru, the Syrian Arab Republic and Tunisia [1]. The disease is associated with poverty and poor living conditions, as well as environmental changes such as deforestation, dam construction, irrigation schemes, and urbanization [1–5].

Leishmaniasis is a climate-sensitive disease as the *Phlebotomus* vectors are thermophilic, requiring warm temperatures for their survival. The developmental stages of these vectors include eggs, larvae, pupae, and adults. The immature stages do not require standing water to complete the life cycle. The hatching of eggs is highly dependent on temperature, with first instar larvae emerging 12 to 19 days after oviposition, pupae in 25 to 59 days, and adults in 35 to 69 days [6]. Laboratory studies have shown that extreme temperatures below 15°C and above 32°C have a negative impact on the fecundity and longevity of the species [7]. The influence of weather variables such as rainfall, relative humidity, soil water stress, evaporation rate, wind speed and El Nino Southern Oscillation on the transmission of leishmaniasis had been evaluated in the past across different endemic settings but the reported associations were inconsistent [8–16]. This heterogeneity depends, in part, on the data and methods used for analysis and the location-specific influences of the climate on vector bionomics of the sand fly species and transmission dynamics of the respective disease entities.

Leishmaniasis has become an increasingly significant public health issue in Sri Lanka. In contrast to the declining disease trends observed in other countries in Southeast Asia, Sri Lanka has been experiencing a steady increase in case numbers of leishmaniasis with an exponential rise in 2018 [17]. Almost all the leishmaniasis clinical cases in Sri Lanka are CL caused by *Leishmania donovani* [18]. The parasite is probably transmitted through the species *Phlebotomus argentipes glaucus*, which demonstrates zoophilic behavior compared to other related species found in India [19, 20]. The continuous upsurge of disease transmission in the country warrants urgent attention to design effective control interventions may be to meet equivalent elimination targets as established in the past for VL in the region. These targets involve reducing the incidence to less than one case per 10,000 population [21, 22]; the targets specified by the WHO roadmap for neglected tropical diseases 2021–2030 [23]. Climate change and related environmental and socio-economic impacts may catalyze the transmission dynamics in future, further aggravating the existing disease burden. The context-specific understanding of the sand fly bionomics including (1) the distribution of the sand fly species in different geographic areas and transmission hotspots in the Island, (2) temporal variation of sand fly abundance and (3) the influence of local weather are important to plan successful control interventions.

## Materials and Methods

### Study areas and meteorological data

**Figure 1:**
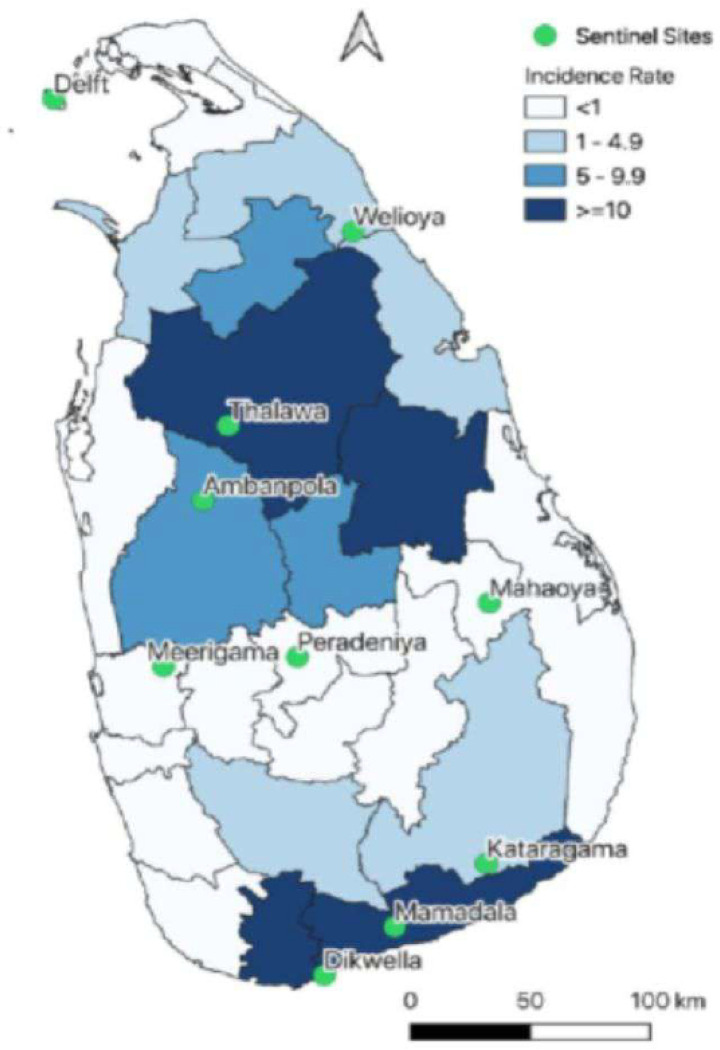
Leishmaniasis annual average incidence rate. Leishmaniasis annual average incidence rate 2018–2020 (per 1000 population) by districts and locations of sand fly surveillance sites in Sri Lanka. Black solid lines in the map represent the boundaries of administrative districts. Green shaded circles indicate the location of long-term sand fly surveillance sites. Source of the base file: https://data.humdata.org/dataset/sri-lanka-administrative-levels-0-4-boundaries.

Sri Lanka is an island with an area of 65,525 km^2^ located between latitudes 5°55’ and 9°51’N and longitudes 79°41 and 81°53’E. The country is divided into four climatic zones based on mainly rainfall, viz. wet zone, intermediate zone, dry zone, and semi-arid zone. The wet zone, located in the southwest part of the island and central hills, receives the maximum rainfall in the country with an annual average of over about 2500mm. The maximum precipitation of rainfall occurs during the southwest (SW) monsoon from May to September and the northeast (NE) monsoon from November to January. The wet zone experiences inter-monsoonal rains too. The dry zone covers most parts of the country and receives an annual rainfall between 1200 to 1900 mm during the NE monsoon with little or no rain for the rest of the year. An intermediate zone that lies between wet and dry zones in the island receives an average annual rainfall of 1500–2500mm whereas the semi-arid zones situated within the dry zone of the country receive an average annual rainfall of 800–1200mm [24, 25]. Considering factors such as the leishmaniasis prevalence, geographical and climatic zones, ten sentinel sites were selected to cover all the nine provinces of Sri Lanka, comprising Welioya (Northern), Delft Islands (Northern), Mahaoya (Eastern), Thalawa (North Central), Ambanpola (Northwestern), Peradeniya (Central), Mirigama (Western), Embilipitiya (Sabaragamuwa), Dickwella (Southern), Kataragama (Uva) (Fig. 1).

Monthly mean rainfall, ambient temperature (minimum and maximum), relative humidity, wind speed, soil temperature (measured at 08:30 and 15:30 hours at 5cm and 10cm), evaporation and sunshine hours data were obtained from the meteorological department of Sri Lanka for the period of March 2018 to February 2020. However, for Delft Island, average values were taken from the past ten years due to limited availability of weather data. The selection of meteorological stations closest to the sampling sites was based on their GPS coordinates. All study settings were selected when evaluating the influence of rainfall, temperature and wind speed. However, based on the availability of selected climatic variables six sites were included for analysis of soil temperature (Welioya, Embilipitiya, Thalawa, Ambanpola, Kataragama, Dickwella), relative humidity (Mahaoya, Embilipitiya, Thalawa, Kataragama, Mirigama, and Dickwella) and evaporation (Welioya, Embilipitiya, Thalawa, Ambanpola, Kataragama and Dickwella) five for sunshine hours (Embilipitiya, Thalawa, Ambanpola, Kataragama and Dickwella).

### Leishmaniasis case incidence

The number of leishmaniasis cases reported to the offices of RDHS (Regional Director of Health Services) of each sentinel-site from March 2018 to February 2020, were obtained from the Epidemiology Unit, Ministry of Health of Sri Lanka [17].

### Sand fly collection

The adult sand flies were collected from March 2018 to February 2020 at ten sentinel sites over a period of twenty-four months. Each site was equipped with two cattle-baited net traps (CBNT) and twenty UV LED CDC traps (BioQuip products, USA). The trapping was conducted for two consecutive days per month, except for Delft Island where trapping occurred only twice a year due to logistic difficulties. The same methodology was used for Delft Island, but the data from these collections were excluded from the analysis due to infrequent sampling. In the case of Peradeniya, the trapping was limited because of challenges in obtaining cattle for CBNTs. The Cattle baited traps used were 10 × 10 Ft in size and a single animal was placed within the trap from 6 pm to 6 am. Sand fly samples were collected at 10 pm and 4 am using a manual aspirator. The collected sand flies were preserved in absolute ethanol and transported to the laboratory for further analysis. All trapped specimens were preserved in absolute ethanol prior to identification.

### Identification of Sand flies

Species identification of collected sand flies was done based on morphological features using standard keys [26].

### District specific characteristics

District-specific characteristics, which could further modify the relationship between weather variability and sand fly density, were selected and obtained from the District Statistics Book published by the Department of Census and Statistics. In addition to the annual average rainfall and temperature, the selected variables were the percentage of land area covered by the forest, paddy fields, inland water bodies, scrub and chena, crops, built environment, and number of livestock and the number of people living in each district. The distribution of these variable among each district is given in the Table S2.

### Statistical analysis

We used a tow-staged hierarchical procedure to evaluate the association between weather variables and sand fly densities. Distributed Lag Non-Linear (DLNM) modelling framework was used to capture the nonlinear and delayed association between weather events and sand fly density. The DLNM method implemented in the R package *dlnm* (version number 2.4.6) uses the concept of creating flexible cross-basis function estimators to capture simultaneously the delayed and non-linear dependencies of the exposure and outcome data [27]. In the first stage, the exposure-lag-response association for each study setting were flexibly estimated using the weather data from the most proximal monitoring station. A quasi-Poisson time series regression model was used to account for the over-dispersion of data and the influence of time-varying confounders.

The common formula for the first stage surveillance site-specific models for weather variables and sand fly density indices is given as

VIi~quasiPoisson(μt)

$$E\left({VI}_{ti}\right)=\beta i+f({Weathre}_{ti},Vardf,lagdf)+s\left(T_{ti}\right.,timedf)$$

Where $E\left(VI_{ti}\right)$ was the expected value for each sand fly index (UV LED CDC traps, CBNT per trap or LT monthly total) in each month (t) in each surveillance setting (i). β was the intercept, and $f\;{(Weathre}_{ti},Vardf,lagdf)$ was the cross-basis function for each weather variable (rainfall, maximum, minimum and mean temperature, soil temperature, relative humidity, sunshine hours etc respectively in each model). The Vardf and lagdf were the corresponding degree of freedom set for weather variables. $s\;{(T}_{ti},timedf)$ was the smooth function of time with the degrees of freedom used to account for the time varying confounders on the outcome.

In the second stage, the surveillance site-specific exposure-response associations were meta-analysed to obtain joint estimates for the country accounting for within and in-between study level variability. The model output was given as a relative risk (RR) estimate calculated with reference to a risk at a predetermined central reference value of the exposure variable. We used a multivariate extension to the Cochrane Q-test of heterogeneity to assess the statistical significance of the heterogeneity of the estimates at each study setting and it was further quantified by using *I^2^* statistics [28]. To examine if the heterogeneity observed could be explained in part by MOH division-specific characteristics (as given in Table S2), we extended this second-stage analysis by regressing these variables in a meta-regression framework provided in the *mvmeta* package. The positive or negative moderator effects of the division-specific variables on the exposer-response associations were predicted at the lower (25th ) and the higher (75th percentile) ends of the range of their values. The statistical significance of the moderator effect was tested by using Wald test statistics.

The models were evaluated with the quasi-Akaike information criterion (q-AIC). We used *mvmeta* package (version number 1.0.3) for the second stage multi-variate meta-analysis [29]. All statistical steps were implemented within using R (version 4.1.0) [30]. A thorough description of the model building and the selection procedure including definition of the cross-basis functions with respect to different knot positioning and model selection procedure is reported in the supplementary appendix (Table S3 and Table S4). The lowest q-AIC values observed for UV LED CDC indicate the better model fit compared to CBNT and LT monthly total for all weather parameters. Therefore, we selected LT per trap for our primary analysis and the results were compared with the other two indices where relevant (Table S3 and Table S4).

## Results

### Sand fly species composition and sex ratio

*Phlebotomus argentipes* was the predominant species, accounting for a total of 38,594 sand flies (34,348 males and 4,246 females), which constituted 99% of the total sand flies captured (Table S1). The remaining sand flies belonged to the *Sergentomyia* genus. The male-to-female ratio in the total sand fly collection was approximately 1:8.09, indicating that there were approximately eight times more males than females (Table S1). A total of forty-eight cattle-baited traps and 960 light traps were used for the collection of *Ph. argentipes* during the study period (Table S1). The male-to-female ratio of *Ph. argentipes* varied depending on the trap type, with a ratio of 10:1 in the cattle-baited traps and 2:1 in the light traps (Table S1).

### Spatial and temporal dynamics

The spatial densities of *Ph. argentipes* captured were highly heterogeneous and variable. Based on the density the sites were arbitrarily classified into High > 2500, Mid 1500–2500 and Low < 1500 zones. Mamadala, Dickwella and Ambanpola were within the high sand fly density zone whereas Kataragama, Mirigama and Thalawa were in mid sand fly density zone and Welioya and Mahaoya were within the low sand fly zone. The sand fly densities however, did not correlate with the leishmaniasis incidence in these areas (stats analysis figure e.g. correlation coefficient / p value > 0.05).

There was temporal variation of sand fly collections in Delft islands also with an apparent negative association with rainfall. While there was a significantly high sand fly collection in April 2018 with low rainfall the collections were poor during the month of October with high rainfall (Fig. S1).

### Exposure-lag-response associations between weather variables and leishmaniasis vector indices

The overall pooled results suggested that rainfall, maximum temperature (ambient), soil temperature measured at 10cm at 8.30am, sunshine hours, mean relative humidity, evaporation, and wind speed were associated with leishmaniasis vector activity (as measured by the UV LED CDC traps) at different lag dimensions across all study settings. The divisional heterogeneity of each exposure response relationship derived by the second stage multi-variate meta-analysis is shown in Table S4. The heterogeneity of the exposure response relationship among study locations was shown to be significant (at a p-value of 0.05) for rainfall, soil temperature 10cm at 08.30 at lag of 2, maximum temperature (ambient) lag 2, and relative humidity. For other variables, the exposure-lag-response associations was found to be homogenous across study settings.

### Rainfall

**Figure 2:**
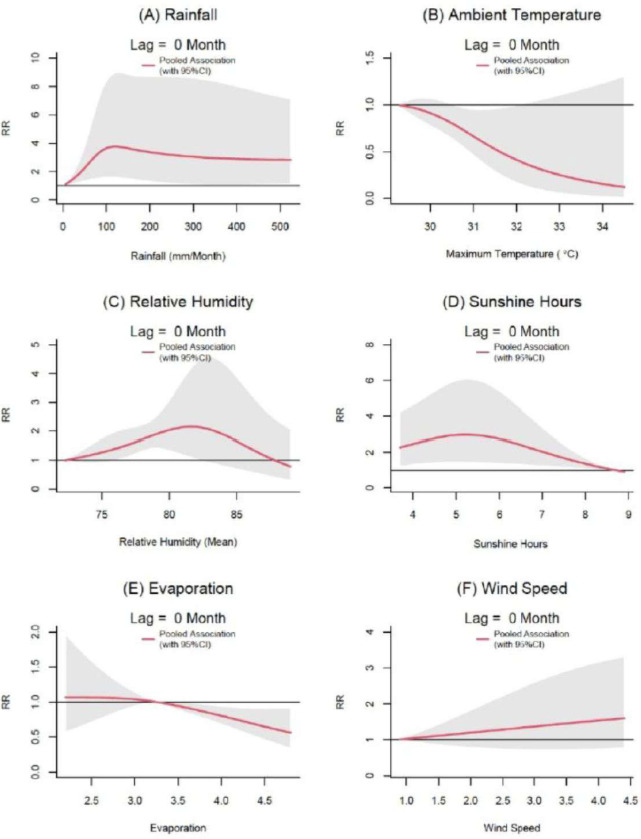
Relative risk (RR) of leishmaniasis vector activity. Relative risk (RR) of leishmaniasis vector activity (measured by UV LED CDC traps) by rainfall (A), ambient temperature (maximum temperature) (B), average relative humidity (C), sunshine hours (D), evaporation (E) and wind speed (F) at a lag of 0 months. The exposure-response functions at lag of 0 month were predicted from the pooled exposure-response function obtained from the meta-analysis for all surveillance sites in Sri Lanka, 2018–20. Shaded areas are 95% CIs. Relative risks were calculated with reference to the risk at a rainfall value of 0 mm per month, maximum temperature of 29.3°C, average relative humidity of 72.25, average evaporation of 3.3mm and wind speed of kmh^−1^. The most important lags for each exposure variable were selected for presentation. The full spectrum of exposure-lag response associations is given in the appendix (pp 7–14).

Rainfall is associated with the risk of increasing leishmaniasis vector activity measured by UV LED CDC trap at lag of 0 (Fig. 2A). With reference to the risk at a rainfall value of 0, the increasing rainfall was associated with the increasing RR of sand fly activity up to 120 mm per month reaching a maximum RR of 3.76 with a 95% CI of 1.58 to 8.96. Thereafter, the RR was observed to decrease with increasing rainfall up to the extreme rainfall value of 524mm per month (RR of 2.83 with 95% CI of 1.12 to 7.14). When the lags are increasing the exposure-response associations became less obvious. The full spectrum of association from lag 0 through lag 3 is given in the Fig. S3.

### Ambient Temperature

At the lag of 0 month, the increasing ambient temperature (maximum temperature) reduced sand fly activity (Fig. 2B). With reference to the lowest temperature value in the range (29.3°C), the sand fly activity appeared to be reduced by each unit increase in the temperature. The associations were statistically significant between 30.6 °C to 32 °C and the minimum relative risk observed at the temperature value of 34.5 °C was 0.12 (95%CI; 0.01 to 1.3). Furthermore, increase in the lag up to two months increased the RR but the associations were not statistically significant (Fig. S5).

### Relative humidity

With referent to the minimum RH value of 72.25, the relative risk of vector activity appeared to increase with increasing RH up to 82 (2.14; 95% CI = 1.04 to 4.38) at lag of 0 month (Fig. 2C). The RR however, decreased with further increase in RH. The association appeared to correlate negatively at a lag period of 2 months having a statistically significant reduction in risk (0.58; 95% CI = 0.37 to 0.91) at a RH value of 80 (Fig. S4).

### Sunshine hours

With reference to the minimum duration of daily sunshine hours of 8.7, the risk of vector activity appeared to increase with decreasing sunshine hours at a lag of 0 months. The maximum relative risk observed (2.93; 95% CI = 1.43 to 6.0) was at 5 hours of sunshine per day (Fig. 2D). When the daily average sunshine hours further reduced, the relative risk of vector activity also appeared to decrease to a lesser extent when compared to increasing sunshine hours. A similar pattern was observed at the lag of 1 month. The association was not statistically significant with a further increase in lags (Fig. S8). The observation was homogeneous across all the settings as suggested by the non-significant Q test (2.77, p-value = 0.950).

### Wind speed

Increasing wind speed appeared to increase the risk of vector activity at the lag of 0 month. However, the associations were not statistically significant up to a lag of 3 months. At the lag of 3 months, the increasing wind speed appeared to increase the relative risk of LT Per trap (Fig. 2e and Fig. S10).

### Soil temperature

**Figure 3:**
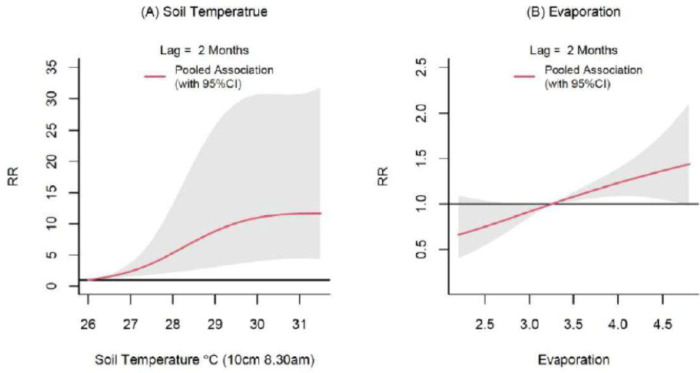
Relative risk (RR) of leishmaniasis vector activity by soil temperature. Relative risk (RR) of leishmaniasis vector activity (measured by UV LED CDC traps) by soil temperature measured at 8.30am at 10cm below the surface (panel A) and evaporation (panel B) at the lag of 2 months. The exposure-response functions at each lag were predicted from the pooled exposure-response function obtained from the meta-analysis for all surveillance sites in Sri Lanka, 2018–20. Shaded areas are 95% CIs. Relative risks were calculated with reference to the risk at a soil temperature of 26°C. The full spectrum of the associations is given in supplementary figures (S13-S14).

Soil temperature measured at 8.30 am at 10cm below the surface was statistically significantly associated with leishmaniasis vector activity (Fig. 3A). The risk of vector activity started to increase at the lag of 1 month and reached its maximum at the lag of 2 months before reducing towards the lag of 3 months (Fig. S6). The maximum risk observed at the lag of 2 months was to be of 11.6 (95% CI; 4.38 to 30.76). Similar to the ambient temperature the RR of sand fly activity decreased with increasing soil temperature at the lag of 0 months. With reference to the risk estimated at 26°C, the lowest relative risk was observed to be 0.12 (95% CI; 0.03 to 0.40) at the soil temperature value of 31°C. At the lag of 0 months, the observed reduction in the risk of vector activity was homogeneous across study settings as suggested by the non-significant Q test. The heterogeneity was statistically significant at a lag of 2 months (Table S4).

### Evaporation

With reference to the evaporation value of 3.25 (which was the median evaporation value observed when averaged across all the settings) the risk of vector activity appeared to decrease with increasing evaporation at a lag of 0 months when the evaporation value exceeded 3.6 (Fig. S9). The minimum relative risk observed (0.56; 95% CI = 0.92 to 0.34) was at an evaporation value of 4.8. An opposite pattern was observed at the lag of 2 months where the RR appeared to increase with increasing evaporation (Fig. 3B). The association was not statistically significant with a further increase in lags. The observation was homogeneous across all the settings as suggested by the Q test (11.2, p-value = 0.339).

### Moderator effects of division-specific factors

Statistically significant moderator effects for the division-specific factors were observed only for the rainfall and soil temperature associations. The association between rainfall and sand fly vector activity was influenced by the divisional variation of the maximum temperature explaining the significant proportion of heterogeneity between study sites, reducing the I^2^ from 49.0 to 37.8 (Table S5). The Figure S11 shows that at the lower temperatures at its 25th percentile value (29.9 °C) positively influence the rainfall and sand fly association causing a higher RR for unit increase in rainfall. In contrast, higher temperatures (33.0°C) which represent 75th percentile of the range had a negative moderator effect (Fig. S11). Lower forest cover and a higher number of people living in the district appeared to have a positive moderator effect on the RR of sand fly vector activity predicted by the soil temperature (Table S6 and Fig. S13 and S14). Annual average rainfalls less than 1200mm (Dry zone) appeared to increase the RR of sand fly activity predicted by rainfall, soil temperature, relative humidity and, sunshine hours (Fig. S13–17).

## Discussion

The aim of the study was to quantify the effect of climatic variables on sand fly vector activity in selected sentinel sites that represent all geographical and climatic zones in Sri Lanka. The temporal variability of sand fly densities was investigated over a period of 24 months in association with the concurrent weather variables viz. mean rainfall, ambient temperature, relative humidity, wind speed, soil temperature, evaporation and sunshine hours. Vector abundance varied with rainfall, soil temperature, maximum temperature and relative humidity either at real time or with a time lag.

The high attractiveness of sand flies to cattle may be attributed to their preference for animal blood, which is enhanced by its greater body size and CO_2_/odour output, and the availability of the cattle for a sustained and successful blood feed [31, 32]. The densities of sand flies tended to differ based on the climatic zone in which the sentinel sites were located. Among the study sites, Kataragama, Mamadala, and Dickwella (in the dry climatic zone) exhibited higher densities of *Ph. argentipes*. In contrast, Ambanpola and Mahaoya (in the intermediate zone) and Welioya (in the dry zone) had lower sand fly collections.

Previous studies conducted in Sri Lanka have also reported *Ph. argentipes* as the predominant species of sand flies [20, 33, 34]. However, this current study is significant in that it demonstrates the widespread presence of *Ph. argentipes* in all sentinel sites, including Delft Island. The sex ratio of sand flies collected in this study was significantly biased towards males in the genus *Phlebotomus*. This is a known phenomenon where male flies are attracted in large numbers to traps containing female flies [35].

There was no correlation observed between sand fly density and the incidence of leishmaniasis cases recorded during the years 2018–2020 [36, 37]. However, this finding may not be surprising since leishmaniasis is a chronic disease, and the manifestation of symptoms typically occurs months or even years after exposure. Additionally, factors affecting the transmission of infection are multifactorial and include vector abundance, among other variables [38].

We found that moderate rainfall events (up to 120mm per month) increased sand fly activity by 3.74 times in the same month. The ability of moderate rainfall to create suitable environmental conditions for adult sand flies to become active, seek blood meals, and [39] oviposition may explain this observation. In contrast, during extreme rainfall, the environmental conditions may become unfavorable for these delicate insects to be active outdoors. Once averaged across all nine study settings, the increasing ambient and soil temperature at lag zero month negatively correlated with the sand fly activity reducing the relative risk below one. Similarly, laboratory experimental studies have found that increasing temperatures more than 32°C was associated with higher mortality rates (around 72%) of adult sand flies [40]. This observation was further supported by our findings of the statistically significant positive moderator effect of lower temperature (29.9 °C or the 25th percentile value for all study settings) and the negative moderator effect of higher temperature (33.0 °C or the 75th percentile value) on the rainfall and sand fly association (Fig. S11 and S12). This observation indicates that a unit increase in rainfall increases the risk of sand fly activity in the areas with low temperatures. However, the soil temperature at a lag of two months was associated with a statistically significant increase in the risk of sand fly vector activity. The studies have found that complete egg to adult development of the sand fly species was temperature dependent and ranged from 27.89 (+/− 1.88) days at 32°C to 246.43 (+/− 13.83) at 18°C [41]. This time lag between oviposition and emergence of adults correlates with the observed time lag of two months found between the soil temperature and sand fly abundance in all Sri Lankan study settings, which might be well within the favourable range for egg hatching and larval development. Exposure to the optimal soil temperatures two months ago may have produced a large number of adults attracted by the light traps at the time of surveillance. Furthermore, the positive moderator effect of the higher number of population and low forest cover may indicate the higher abundance of sand fly breeding close to the human dwellings. Human interactions with the environment may create suitable breeding grounds due to the associated high prevalence of rodents, livestock shelters and irrigation canals [39, 42]. Increasing mean RH up to 82 during the same month of surveillance may have created a suitable environmental condition for the sand fly to be active in the environment. The negative effect of the evaporation and the higher RR observed for low number of sunshine hours at lag zero signify the relative inactivity of the sand flies during extremely dry conditions with a low RH. A positive and favourable interaction of the weather variables in the dry zone may be more conducive for the sand fly vectors to thrive and transmit the *Leishmania* parasite.

Among the factors investigated, wind speed has the potential to influence the dynamic behavior of sand flies, particularly in terms of gene flow between populations without geographical barriers. This gene flow can facilitate the transfer of genes that promote sand fly survival, such as insecticide resistance genes [34], which can have negative implications for vector control programs. However, the present study did not find any relationship between sand fly density and wind speed. Future research endeavors aimed at assessing the impact of environmental factors [43, 44], such as wind speed, could provide valuable insights for anticipating public health challenges and developing strategic plans for disease control.

## Limitations

The data analysis of the study was limited to less than nine sites for all variables except rainfall and temperature because of lack of climate data available at close proximity to the given site. Furthermore, the density of sand flies in Delft Island was monitored only bi-annually due to logistical constraints and was analysed against the climatic data recorded off the Jaffna peninsula (40 KM away from Delft), which has the nearest meteorological station, which is also a limitation of this study. Measures of microclimatic variables however, do not seem to affect vector density [42]. Furthermore, the macroclimate temperature and rainfall recorded at the closest meteorological stations have correlated negatively with vector densities elsewhere [42]. The inclusion of ground-level climate data is preferable due to their comparative reliability to remote sensing data. The two-staged hierarchical approach we adopted is likely to have overcome this limitation to a certain extent by its ability to pool the evidence of multiple study settings making the findings more robust.

### Implications of the findings for vector control, disease control, climate change and meeting WHO 2030 targets for NTDs.

The findings are of significance in forecasting vector abundance and designing of effective strategies to curtail leishmaniasis transmission in a given setting during an era of escalating concern over climate change. This study while adding to the evidence linking leishmaniasis incidence with changes in environmental factors, provides novel information on the likely effect of selected environmental factors on developing sand fly stages, with resultant lag effect observed on adult sand fly abundance. This observation may be used in establishing an early disease warning system for local populations to aid control. Favourable climatic conditions in terms of temperature, rainfall and humidity experienced by local sand fly population enhance leishmaniasis outbreaks. A temperature range between 29.9 and 33.0 oC, a humidity level up to 82% and the presence of moderate rainfall (up to 120mm per month) were optimal parameters for the development, the longevity of sand flies and transmission of leishmaniasis consequently. Furthermore, the results presented here suggest that the state of vegetation may also play a role in establishing favorable environmental conditions for leishmaniasis across Sri Lanka. Overall, these findings, demand a regionally-coordinated strategic plan to address the apparent threat of increasing RR of leishmaniasis particularly in the face of changing climatic factors. Such an effort may increase the chance of achieving the WHO 2030 targets for effective control and elimination of NTDs in the region.

## Conclusions

The sand fly abundance correlates with environmental parameters such as rainfall, soil temperature, maximum temperature, and relative humidity either at real-time or with a time lag. The findings can be used for sand fly density predictions and the design of effective strategies for leishmaniasis transmission in a given setting. To further enhance our ability to predict disease outbreaks, it is essential to combine these environmental findings with epidemiological and demographic data, as well as robust surveillance systems. This holistic approach, incorporating a comprehensive understanding of the environmental factors and the ecology of leishmaniasis, will enable the refinement of existing approaches and the development of more accurate disease outbreak predictions.

## Data Availability

Climate data collected for the study is available upon request from the Department of Parasitology, Faculty of Medicine, University of Colombo, Colombo, Sri Lanka and Department of Research and Evaluation, National Institute of Health Sciences Kalutara, Sri Lanka

## Abbreviations

DLNM: Distributed Lag Non-Linear
VL: Visceral leishmaniasis
MCL: Mucocutaneous leishmaniasis
CL: Cutaneous leishmaniasis
WHO: World Health Organization
RDHS: Regional Director of Health Services
CBNT: Cattle-baited net trap
UV light trap: Ultraviolet light trap
NE: Northeast
SW: Southwest
LT: Light trap
RR: Relative risk
MOH: Medical officer in Health
q-AIC: Quasi-Akaike information criterion
CI: Confident Interval
RH: Relative humidity
CO_2_: Carbondioxide
NTDs: Neglected Tropical Diseases
